# The African Network for Evidence-to-Action on Disability: A role player in the realisation of the UNCRPD in Africa

**DOI:** 10.4102/ajod.v3i2.86

**Published:** 2014-06-04

**Authors:** Rachel Kachaje, Kudakwashe Dube, Malcolm MacLachlan, Gubela Mji

**Affiliations:** 1 Southern Africa Federation of the Disabled, Bulawayo, Zimbabwe; 2 African Decade for Persons with Disabilities, Pretoria, South Africa; 3 Centre for Global Health and School of Psychology, Trinity College, University of Dublin, Ireland; 4 Centre for Rehabilitation Studies, Stellenbosch University, South Africa

## Abstract

This *African Journal of Disability* supplement focuses on papers presented at the third AfriNEAD Symposium in 2011. In this closing editorial, we want to give an overview of the rationale and major modes of operation of the African Network for Evidence-to-Action on Disability (AfriNEAD) with special focus on recommendations made at the 2011 AfriNEAD Symposium. AfriNEAD is guided and informed by the United Nations Convention on the Rights of Persons with Disabilities (UNCRPD) for its research themes. The issues that emerged from AfriNEAD 2011 ranged from children and youth with disabilities; education across the lifespan; economic empowerment; the development process in Africa; health, HIV and AIDS and community-based rehabilitation; holistic wellness; to research evidence and utilisation. Disability-related stigma, the value of emancipatory research and the need to recognise a broader scope of valid methodologies were also highlighted.

## Introduction

People with disabilities in Africa are not yet experiencing meaningful change in their quality of life, access to equal rights and level of community integration, despite a favourable policy environment in some countries and a supportive research evidence base. Real change and social development are still required to realise a truly inclusive society (Mji *et al*. 2009). Inclusive policies that facilitate all people to access equal human rights will assist in unlocking the potential of people with disabilities to make contributions to the development agenda in their various countries. The vision of the African Network for Evidence-to-Action on Disability (AfriNEAD) is premised on the need to utilise existing and new knowledge and evidence from a diverse range of research topics and sources.

AfriNEAD facilitates a multidimensional, intersectoral, interactive forum that focuses on how the evidence that is generated by disability researchers is translated to action (Mji *et al*. 2011). AfriNEAD also affords disability researchers exposure to the complexities of the issues of equity within the disability field and the need to see disability as a social and developmental issue. AfriNEAD is a forum that builds and sustains relationships, partnerships and cooperation between researchers and users of research evidence and knowledge. The forum provides a platform where research results are better able to target and influence policy and practice – AfriNEAD lives in the research-policy-practice ‘gaps’ seen as ‘problems’ in other fora. Realising that such ‘problems’ present the greatest opportunities for learning and enacting learning is the driving force behind AfriNEAD.

## Specific AfriNEAD objectives

AfriNEAD was inaugurated at the 2007 Symposium, where five objectives were tabled to achieve its goal:

1. develop a network to facilitate and coordinate research dialogue from evidence-to-action
2. establish AfriNEAD working groups that will identify action pathways and produce best evidence-to-action practice guidelines
3. implement *Research on evidence-to-action in disability* (READ) for Africa, a methodological profiling of own local situation regarding disabled people
4. hold the AfriNEAD Symposium, a platform essential for the dissemination and utilisation of research, at least once every three years
5. link disability, academia, business, government and civil society to develop a DABGC (Disability, Academics, Business, Government and Civil Society) Consortium.

Each of these objectives is briefly reviewed below before considering recommendations made at the 2011 Symposium.

### Develop a network to facilitate and coordinate research dialogue from evidence-to-action

AfriNEAD provides space for dialogue. Dialogue has been facilitated by establishing a network whose primary objective is to explore effective ways of utilising knowledge as a basis for planning action. The dialogue takes place between Disabled People Organisations (DPOs), academics, activists, practitioners, employers and civil servants from different parts of the African continent – all focused on the need to effect positive change in the quality of lives of people with disabilities. In this space, unlike in formal debates, we do not look for winners and losers; rather, it provides a medium for finding common ground, sometimes by addressing mistrust and misconceptions. Whilst the symposium indeed sought to make recommendations and decisions, dialogue provided the means to achieve consensus.

Importantly, AfriNEAD is not responsible for doing research itself but rather for facilitating and coordinating an enabling environment for others to do research in relevant areas, helping to identify gaps and to fill them with useable ideas.

### Establish AfriNEAD working groups to produce best evidence-to-action practice guidelines

AfriNEAD’s aim was to establish interdisciplinary and multiperspective ‘working groups’ tasked with identifying action pathways and producing best evidence-to-action practice guidelines which would be published on the AfriNEAD webpage. Work groups are essential platforms for dialogue and debates, thus engendering change processes with principles of participation, consensus building and joint decision making within AfriNEAD. The approach allows ‘cross-fertilisation’ of ideas, reduced bias, increased risk taking, high commitment, improved communication and overall, we hope, achievement of better solutions.

### Implement READ for Africa

The AfriNEAD network will work with member countries to develop a methodology for profiling their own local situation regarding persons with disabilities, the services available to them, the research being conducted and areas needing investigation, and to identify facilitators and barriers for turning evidence into action. It is anticipated that countries will submit their ‘state of our nation’ analysis for publication in an e-book available on the AfriNEAD webpage. This publication, entitled *Research on evidence-to-action in disability for Africa* (*READ for Africa*), will be an important advocacy document. The publication will act as a dissemination tool for the network’s activities, will provide opportunities for researchers to contribute to a reputable forum of high status and will act as a mechanism to link with research support.

### Host a tri-annual AfriNEAD symposium

Another platform that is essential for dissemination and utilisation of research is the AfriNEAD Symposium, held at least once every three years. This three-day Africa-wide symposium focuses on specified themes of interest to network members in order to assess progress on evidence-to-action and to consolidate and strengthen dialogue The symposium is hosted on a rotation basis by member countries, with each event producing a collection of papers to be published as a Special Issue of a suitable international journal. The first special issue focusing on the 2007 AfriNEAD Symposium was published by the well-established international journal *Disability and Rehabilitation*. A paper in the respected *Disability and Society* subsequently outlined the outcomes of the 2009 AfriNEAD Symposium (Mji *et al*. 2011). The *African Journal of Disability* is the second journal since the inauguration of AfriNEAD in 2007 to table a special issue focusing on the AfriNEAD Symposium. Indeed the impetus for this journal *– African Journal of Disability* – comes, at least in part, from the enthusiasm generated by energetic researchers meeting through AfriNEAD.

### Develop a DABGC Consortium

There is a need to bridge the gap between all critical stakeholders of AfriNEAD. The desire for a mutual exchange of skills, knowledge and resources led to the development of a DABGC Consortium, linking disability, academia, business, government and civil society, proposed as a platform for engaging sectors of the consortium that have a direct impact on and association with the world of people with disabilities. The proposed focus of the DABGC Consortium will be to address the gaps that exist between the stakeholders’ worlds, especially those of business and people with disabilities – for instance by developing plans to facilitate the entry of qualified people with disabilities into the workplace, and supporting the vision and goals of AfriNEAD through use of combined resources.

## Using the UNCRPD as a framework for AfriNEAD research activities

The United Nations Convention on the Rights of Persons with Disabilities (the Convention or UNCRPD hereafter) obligates state parties to bring people with disabilities into the mainstream of society and development. It is a legally binding document for those countries that have ratified it, and provides a universal standard of human rights for all persons with disabilities regardless of the country in which they are in (Dube 2009).

During preparations for the second symposium, the coordination committee resolved that to improve its relevance to its key stakeholders (people with disabilities), AfriNEAD will use the Convention as a framework that will guide the network on how to organise presentations and discussions for the symposium. The articles of the UNCRPD were compressed into seven themes. The calls for abstracts have been based on these themes, with a scientific committee allocating the abstracts into these seven themes. They are:

- Children and youth with disabilities
- Education: early to tertiary
- Economic empowerment
- Development process in Africa: poverty, politics and indigenous knowledge systems
- Health, HIV/AIDS and community-based rehabilitation
- Holistic wellness: sport, recreation, sexuality and spirituality
- Research evidence and utilisation and making clear recommendations for policy and practice.

Below we present the recommendations made at the 2011 AfriNEAD Symposium relating to these seven themes.

## 2011 AfriNEAD Symposium recommendations

The 2011 Symposium comprised representatives from 18 African countries and seven countries beyond Africa, all incorporating government, researchers, DPOs, civil society and non-governmental organisations (NGO), and business. Based on the seven themes extrapolated from the UNCRPD, the following recommendations were made at the 2011 AfriNEAD symposium.

### Children and youth with disabilities

Presenters from this theme highlighted the value of conducting needs assessment for children with disabilities in Africa. Where possible, children should be included and informed (ensure appropriate informed consent and assent) in most of the decisions that affect them. At all times and where possible, self-representation of children should be encouraged. A communication freeway between parents and service providers, as well as researchers and policy makers, should be developed. Parents’ experiences should be valued. Capacity building needs to be done across all levels. There should be knowledge translation processes between researchers, parents and children with disabilities underpinned by the principle of family centredness.

### Education: Early to tertiary

This theme focused on three critical areas:

- **Educator training** (teachers and lecturers), with emphasis on curriculum adaptation and inclusive assessment, policy awareness, and a paradigm shift from focus on only early childhood education to focus on continuing higher/life-long learning.
- **Inclusion of disability issues in the curriculum across disciplines in higher education**, including training all future professionals, policy makers and researchers in disability issues so that they can include disability at all levels, including the classroom.
- **Responsive research**, including defining a research agenda according to practical needs and best practice, children’s and families’ experiences, implementing policy, developing reliable databases (and knowledge management systems) and highlighting education as a gateway to success.

Three strategies were tabled to address the above:

- following up on issues of discrimination and exploring ways of taking legal action
- advocacy and lobbying – working with policy makers, DPOs and other relevant stakeholders
- writing papers based on the three key issues listed above.

### The development process in Africa: Politics, poverty and indigenous knowledge systems

In this theme, issues of poverty and access – though relevant to everyone in society – were highlighted as relevant to people with disabilities in particular as they are more likely to be excluded. Evidence demonstrates a significant association between poverty and disability. When addressing needs of people with disabilities, African methods of governance, such as tribal authority and imbizo, should be considered versus ‘modern’ government structures. However, traditional leaders must also be convinced that some cultural beliefs about people with disabilities are simply myths, stigmatising and negatively impacting those with disabilities.

There is a need for specifically designed advocacy programmes targeting traditional leaders in rural areas to combat culture-based violent discrimination against people with disabilities. Health service providers need to understand the barriers that people with disabilities face if service delivery is to be equitable and inclusive. On the other hand, people with disabilities should demand their health rights as enshrined in different statutes. DPOs should link with service providers and form networks with the law fraternity to investigate policies and legal frameworks that will ensure inclusion of people with disabilities in policy development processes. AfriNEAD needs to work with research institutes and national bureaux of statistics to make disability visible in the monitoring of national development plans and the Millennium Development Goals.

### Economic empowerment

Three recommendations were made relating to this theme:

- There is an urgent need for a paradigm shift from traditional career guidance to career construction in the way persons with disabilities perceive lifelong career choices by utilising the concept of entrepreneurship.
- There is a need to decentralise the provision, design, repair and maintenance of assistive devices and empower disabled people to be the custodians of this process through support from government and other stakeholders.
- There is a need for African governments to domesticate the UNCRPD to ensure the right to equal access to transportation, information, services and facilities. Equal access can be achieved through the engagement of professionals and experts in the development of legislation, access guidelines and operationalising policy in making the environment accessible for all.

### Health, HIV and AIDS and community-based rehabilitation

The following three recommendations were made with regard to this theme:

- Building a critical mass within mainstream society on disability issues, including mainstream human rights organisations such as Amnesty International and Human Rights Watch.
- Investigating the discrepancy between policies and their implementation, and using this evidence supported by key quantitative data to advocate at government level.
- Motivating and lobbying governments to adopt community-based rehabilitation as part of their national rehabilitation policy.

### Holistic wellness: Spirituality, sport and recreation

The focus for this theme was improved accessibility to all support systems that will facilitate people with disabilities to engage in activities that enhance holistic wellness, such as sport, recreation and spiritual activities. Appropriate assistive devices (e.g. wheelchairs, prostheses, orthotics, hearing aids and accessible transport) are essential to achieve success in this regard. Disability is not necessarily a hindrance; rather, it can it can illustrate to society how people can successfully cope with challenges. However, it is essential to improve public education on disability issues and adaptation, and develop human resources to support people with disabilities. In all of this it is important to understand the context and take into consideration indigenous knowledge systems, including local and global issues.

### Research evidence and utilisation

Three key issues were highlighted for this theme:

- Evidence-based practice requires an alignment between research, participation and feedback from the community, for effective intervention and/or practice to flourish.
- There is a need to explicitly recognise and value alternative forms of gathering information, to complement the more established and rigorous scientific methodologies. There is a plethora of rich information that needs to be collected and structured for recognition by academic circles so that it too can inform policy and be used to address identified gaps.
- Once research is put into action, there needs to be ongoing monitoring and evaluation to ensure that practice remains linked to evidence, informed by and appropriate to the cultural setting, to promote continued effective outcomes.

## Discussion

AfriNEAD is a network that has managed to bring key stakeholders to the table to discuss how research evidence can be used as a tool to facilitate the realisation of rights for people with disabilities in Africa. The recommendations made at the 2011 Symposium outlined above are the collective and consensual products of participants in the meeting, not just the authors of this paper. Aligning research themes with the UNCRPD adds credibility to AfriNEAD in its quest for using the network as a platform to debate the impact of research evidence on realising the rights of people with disabilities in Africa. The papers presented in this supplement also emerged from the seven themes extrapolated from the UNCRPD.

AfriNEAD represents local research capability which finds expression when the researcher can facilitate public debate amongst researchers, non-researchers, policy makers and other end-users, highlighting the multiple interests and positions of power in society (Nair & Menon 2002) and also in international aid efforts (MacLachlan, Carr & McAuliffe 2010). AfriNEAD has thus based its modus operandi on research that is both emancipatory and demand-led. The advantages that flowed from this approach are that it:

- facilitates active participation of both demand-side and supply-side entities
- provides an opportunity to work with the real needs of people with disabilities
- makes it possible to vigorously interrogate the policy formulation and implementation processes
- provides real opportunities for political and administrative buy-in and implementation.

In reporting on the 2011 Symposium it strikes us that one of the greatest barriers to equalisation of opportunities for people with disabilities are negative attitudes. Symposium delegates recommended that governments develop and implement programmes to address negative attitudes, myths, beliefs and discrimination at all levels associated with the stigma of being disabled. Holistic programmes such as community-based rehabilitation strategies and HIV and AIDS programmes, developed and funded by governments, could be used as platforms to address such attitudes. Such programmes should be based on the principles of inclusion, empowerment and sustainability. Finally, ignored and hidden impairments, such as intellectual disability, should be made a priority within an inclusive holistic approach to disability.

## Conclusion

This closing editorial provides a review of some of the recommendations of the 2011 AfriNEAD Symposium. A key focal point in the recommendations of the symposium is the need for a paradigm shift regarding the way researchers and research users have traditionally interacted. For people with disabilities, active participation in research and its resultant utilisation is crucial as historically they were simply perceived as ‘research subjects’. Thus the 2011 Symposium placed emphasis on building the capacity and confidence of people both with and without disabilities, who are researchers themselves or are participants in various stages of the disability research cycle.
